# Ocular Safety and Toxicokinetics of Bevacizumab-bvzr (Zirabev), a Bevacizumab Biosimilar, Administered to Cynomolgus Monkeys by Intravitreal Injection

**DOI:** 10.1089/jop.2022.0059

**Published:** 2023-04-04

**Authors:** Marjorie A. Peraza, Susan Hurst, Wenhu Huang, Bernard S. Buetow, Andrew J. Lickteig, J. Dan Lavach, Denzil F. Frost, Margaret E. Collins, Rani S. Sellers, Diane Matsumoto Smith

**Affiliations:** ^1^ Drug Safety Research and Development, Pfizer Inc., Cambridge, Massachusetts, USA.; ^2^ Biomedicine Design, Pharmacokinetics, Dynamics, and Metabolism, Pfizer Inc., Groton, Connecticut, USA.; ^3^ Drug Safety Research and Development, Pfizer Inc., San Diego, California, USA.; ^4^ Charles River Laboratories, Inc., Reno, Nevada, USA.; ^5^ Drug Safety Research and Development, Pfizer Inc., Pearl River, New York, USA.

**Keywords:** bevacizumab biosimilar, ocular safety, intravitreal injection, nonclinical

## Abstract

**Purpose:**

Bevacizumab-bvzr (Zirabev^®^), a recombinant humanized monoclonal antibody targeting vascular endothelial growth factor and a biosimilar to bevacizumab, is approved for intravenous administration for various indications worldwide. The objectives of this study were to evaluate the ocular toxicity, systemic tolerability, and toxicokinetics (TKs) of bevacizumab-bvzr following repeat intravitreal (IVT) injection to cynomolgus monkeys.

**Methods:**

Male monkeys were administered saline, vehicle, or bevacizumab-bvzr at 1.25 mg/eye/dose once every 2 weeks (3 doses total) for 1 month by bilateral IVT injection, followed by a 4-week recovery phase to evaluate the reversibility of any findings. Local and systemic safety was assessed. Ocular safety assessments included in-life ophthalmic examinations, tonometry (intraocular pressure, IOP), electroretinograms (ERGs), and histopathology. In addition, concentrations of bevacizumab-bvzr were measured in serum and in ocular tissues (vitreous humor, retina, and choroid/retinal pigment epithelium) and ocular concentration-time profiles and serum TKs were evaluated.

**Results:**

Bevacizumab-bvzr was tolerated locally and systemically, with an ocular safety profile comparable to the saline or vehicle control group. Bevacizumab-bvzr was observed in both serum and in the evaluated ocular tissues. There were no bevacizumab-bvzr-related microscopic changes or effects on IOP or ERGs. Bevacizumab-bvzr-related trace pigment or cells in vitreous humor (in 4 of 12 animals; commonly associated with IVT injection) and transient, nonadverse, mild ocular inflammation (in 1 of 12 animals) were noted upon ophthalmic examination and fully reversed during the recovery phase.

**Conclusions:**

Bevacizumab-bvzr was well tolerated via biweekly IVT administration in healthy monkeys, with an ocular safety profile comparable to saline or its vehicle control.

## Introduction

Bevacizumab-bvzr (Zirabev^®^), a recombinant humanized monoclonal antibody targeting vascular endothelial growth factor (VEGF) and biosimilar to bevacizumab,^1,2^ has been approved for intravenous (IV) administration for various oncologic indications first by the European Commission in February 2019, followed by other countries worldwide.^3–5^ Bevacizumab binds VEGF and inhibits its activity, thereby blocking VEGF-induced angiogenesis and other downstream effects.^6^ VEGF overexpression has been associated with cancers^7–11^ and in pathological neovascularization in several diseases of the eye, including wet age-related macular degeneration (AMD) or neovascular AMD (nAMD).^12^

nAMD is one of the leading causes of vision impairment and blindness in the elderly.^13^ nAMD is a major cause of central visual loss in the developed world and age-related, affecting 2.2% in the 65–69 years, 15.8% for the 85–90, and 21.2% for people older than 90 years.^14^ Although many therapeutic approaches have been explored, presently the most effective and dominant treatment for nAMD is antibody-based VEGF inhibition therapy with direct intravitreal (IVT) administration, such as Lucentis^®^ (ranibizumab), a recombinant humanized IgG1 monoclonal Fab fragment being one such marketed anti-VEGF drug. Later, another anti-VEGF antibody bevacizumab was shown to have similar efficacy and safety in a 2-year long interventional clinical study.^15^

To support the registration of bevacizumab-bvzr, limited nonclinical studies were undertaken to compare its physiochemical characteristics and functional activity to that of the bevacizumab originator product. These studies included both *in vitro* assays and *in vivo* IV toxicity studies, which included toxicokinetic (TK) analysis.^16^

Based on the high VEGF sequence similarity between human and cynomolgus monkey and evidence of pharmacology (i.e., physeal dysplasia of the distal femur secondary to angiogenesis inhibition in skeletally immature animals) in cynomolgus monkey toxicity studies by the originator,^1,17,18^ the cynomolgus monkey was considered a suitable and pharmacologically relevant species for determining ocular toxicity and TK of bevacizumab-bvzr.^16^ The cynomolgus monkey is a U.S. Food and Drug Administration (FDA)-recommended ocular toxicity species for its similar ocular biological/anatomic structure to the human eye.^19–21^ Therefore, the current study was undertaken to comprehensively assess the ocular tolerability, toxicity, and TK of bevacizumab-bvzr compared with its vehicle and/or saline when administered at a clinically relevant dose and dose volume by bilateral IVT injection in cynomolgus monkeys.

The experimental design of this bevacizumab-bvzr IVT study was consistent with that used to assess the nonclinical ocular toxicity of Lucentis and Byooviz™ (a biosimilar to Lucentis) and was aligned with dosing recommendations of other IVT drugs.^21,22^ For a robust assessment of ocular toxicity and tolerability, study endpoints included in-life ophthalmic examinations, intraocular pressure (IOP), electroretinograms (ERGs), and histopathology of the eyes. Ocular tissue bioanalysis and systemic TK were also evaluated.

## Methods

This research followed the national and institutional guidelines for the care and use of laboratory animals.^23–27^ This research also followed the Association for Research in Vision and Ophthalmology (ARVO) Statement for the Use of Animals in Ophthalmic and Vision Research. Accordingly, bilateral IVT administration was undertaken in the interest of minimizing the number of animals on study; is precedented^21,22^; has a low likelihood of affecting vision; and is not typically associated with adverse effects, particularly when administered by experienced veterinary professionals, as was the case for this study. Prior to conduct, all procedures involving the use of animals in these experiments were under protocols approved by the Institutional Animal Care and Use Committee (IACUC) at Charles River Laboratories.

### Animals and study design

Male cynomolgus monkeys from Mauritius (provided by Charles River Laboratories, Research Model Services, Houston, TX) were 3.4–3.8-years of age, 3.3–5.2 kg, and confirmed tuberculosis negative at the study start. The study included 18 monkeys that were divided into 3 groups (*n* = 6 per group). The total number of animals, the group size, and the number of groups were the minimum required to properly characterize the effects of bevacizumab-bvzr, in line with minimizing use of nonhuman primates (NHPs).^28,29^ Monkeys were group (up to 3) housed under standard conditions (details see Supplementary Data).

Bevacizumab-bvzr was administered once every 2 weeks (total of 3 doses; on Days 1, 15, and 29) by bilateral IVT injection at 1.25 mg/eye (50 μL dose volume) for animals in Group 2 and 3. The dose, dosing frequency, and dose volume aligned with the clinical application of Lucentis and other drugs administered intravitreally.^21,22^

**FIG. 1.**
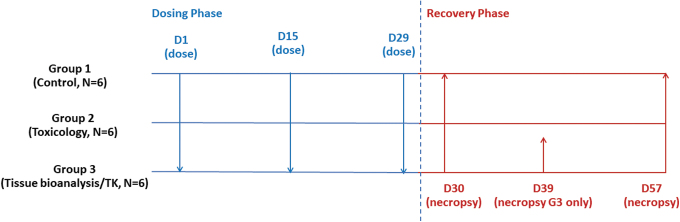
Experimental design. **Treatment administered by IVT injection** Group 1: 0.9% saline (50 μL) into the OS of each animal, vehicle control (50 μL) into the OD of each animal; Group 2: bevacizumab-bvzr 1.25 mg (50 μL of 25 mg/mL), bilateral of each animal; Group 3: bevacizumab-bvzr 1.25 mg (50 μL of 25 mg/mL), bilateral of each animal. Assessment* Predose: Weekly: ***body weights*** Daily: ***mortality, cage-side clinical observations, food consumption*** Predose: ***ophthalmic examinations, tonometry***, ***ERGs, veterinary physical examinations*** Week −2: ***ADA***. Dosing phase: D1: ***TK*** Groups 1–3: predose, 8, 24, 48, and 168 h; ***ADA*** Groups 1–3: 168 h D15:***TK*** Groups 1–3: predose, 8, 24, 48, and 168 h; ***ADA*** Groups 1–3: 24 and 168 h D3, D17: ***ophthalmic examinations, tonometry*** Groups 1–3 Week 4: ***ERGs***, ***veterinary physical examinations*** Groups 1–3 D29: ***TK*** Groups 1–3: predose, 8, and 24 h; ***ADA*** Groups 1–3: 24 h D30: ***necropsy*** Group 1 (*N* = 4), Group 2 (*N* = 4), Group 3 (*N* = 2); ***tissue collection for bioanalysis*** Group 1 (*N* = 1) and Group 3 (*N* = 2) (nonrecovery animals only); ***tissue collection for histology, microscopic evaluation*** Groups 1–2 (nonrecovery animals only). Recovery Phase: D29: ***TK*** Groups 1–3 (recovery animals only): 48, 168, and 336 h; ***ADA*** Groups 1–3 (recovery animals only): 48, 168, and 336 h D31, D39, D49: ***ophthalmic examinations, tonometry*** Groups 1–3 (recovery animals only) D39: ***necropsy*** Group 3 (*N* = 2); ***tissue collection for bioanalysis*** Group 3 (*N* = 2) (recovery animals only) D49: ***veterinary physical examinations*** Groups 1–3 (recovery animals only) D51: ***ERGs*** Groups 1–3 (recovery animals only) D57: ***TK*** Groups 1–3 (recovery animals only): single timepoint; ***ADA*** Groups 1–3 (recovery animals only): single timepoint; ***necropsy*** Group 1 (*N* = 2), Group 2 (*N* = 2), Group 3 (*N* = 2); ***tissue collection for bioanalysis***: Group 1 (*N* = 1) and Group 3 (*N* = 2) (recovery animals only); ***tissue collection for histology, microscopic evaluation***: Groups 1–2 (recovery animals only). *Assessments at each time point are in ***bold italic***. ADA, anti-drug antibody; D, study day; ERGs, electoretinograms; G, group; IVT, intravitreal; OD: right eye; OS, left eye; TK, toxicokinetics.

Ocular tolerability of the vehicle control article [containing excipients only (edetate disodium dihydrate [Avantor, Radnor, PA], polysorbate 80 [Sigma-Aldrich, St Louis, MO], succinic acid [Merck KGaA/EMD Millipore, Darmstadt, Germany], sucrose [Merck KGaA/EMD Millipore, Darmstadt, Germany], and sterile water for injection [B. Braun Medical, Melsungen, Germany], United States Pharmacopeia [USP]^4^) and no active molecule] relative to 0.9% saline (0.9% sodium chloride USP, ICU Medical, Austin, TX) was evaluated under the same dosing regimen and volume by contralateral IVT injection (vehicle in right eye; saline in left eye) to animals in Group 1. The endotoxin levels for vehicle, saline, and bevacizumab-bvzr) were not more than 0.01 EU/mg for each. A 4-week dose-free recovery phase was included to evaluate the reversibility of any findings (Fig. 1).

A staff veterinarian trained to perform IVT injections carried these out. A single injection of sustained release buprenorphine [0.2 mg/kg, subcutaneous (SC) injection; ZooPharm, Laramie, WY] was administered once before each IVT injection. The animals were sedated with ketamine [15 mg/kg, intramuscular (IM) injection; Zoetis, Parsippany, NJ] and dexdomitor (0.06 mg/kg, IM; Zoetis) for the dosing procedure. The eyes were cleansed with Betadine^®^ (5% ophthalmic prep solution; Alcon, Geneva, Switzerland) and rinsed with sterile saline (0.9% sodium chloride; Baxter Healthcare, Deerfield, IL) in preparation of the IVT procedure. Before dose administration, tropicamide (1% ophthalmic solution, 1–3 drops per eye; Akorn, Lake Forest, IL) was instilled in each eye, followed by proparacaine topical anesthetic (0.5% ophthalmic solution, 1–3 drops per eye; Bausch & Lomb, Bridgewater, NJ).

An eyelid speculum was inserted to keep each eyelid open during the IVT injection and the globe was retracted. The injection site was swabbed with Betadine and lightly blotted. The needle (27G) was inserted through the superior temporal sclera and pars plana ∼4 mm posterior to the limbus and directed posterior to the lens and advanced into the mid-vitreous. Each eye received a single-dose injection of 50 μL of saline, vehicle control, or bevacizumab-bvzr (1.25 mg). Injections were slowly administered into the mid-vitreous. Tobramycin (0.3% ophthalmic solution, 1–3 drops per eye; Wintac Ltd., Bangalore, India) was dispensed onto each eye immediately following dosing and for two consecutive days after dosing. Sedation was reversed with Antisedan^®^ (atipamezole; 0.6 mg/kg, IM; Zoetis) (Supplementary Data).

Assessment of toxicity included mortality, clinical (cage-side) observations, body weight, qualitative food consumption, ophthalmic examinations, tonometry, electroretinography, veterinary physical examinations (body temperature, heart rate, respiration rate), and anatomic pathology (microscopic examination of eyes and optic nerves) (Fig. 1). Blood samples for serum TK assessments were collected at predose; 8, 24, 48, and 168 h postdose of the Day 1, 15, and 29 dose; and additionally at 336 and 672 h postdose of the Day 29 dose. Blood samples were also collected for serum antidrug antibody (ADA) assessment at predose Week −2 and at specified times in relationship to the Day 1 dose (168 h postdose), Day 15 dose (24 and 168 h postdose), and Day 29 dose (24, 48, 168, 336, and 672 h postdose).

Ocular tissues [vitreous humor (VH), choroid/retinal pigment epithelium (C/RPE), retina] were collected for bioanalysis from Group 1 (control; *n* = 1 per necropsy on Days 30 and 57, respectively) and Group 3 animals (bevacizumab-bvzr-dosed; *n* = 2 per necropsy on Days 30, 39, and 57, respectively). Necropsies for collection of eyes for histology and microscopic evaluation were conducted on Day 30, 1 day after the last dose (control, *n* = 3; Group 2 bevacizumab-bvzr-dosed, *n* = 4) and following the 4-week recovery period on Day 57 (control, *n* = 1; Group 2, *n* = 2). Animals were sedated with ketamine (10-17 mg/kg) and euthanized by IV injection of pentobarbital (120–150 mg/kg; Vortech Pharmaceuticals Ltd., Dearborn, MI), followed by exsanguination.

### Ophthalmic examinations and tonometry

Ophthalmic examinations and tonometry to measure IOP were performed on Days −11, 3, 17, 31, 39, and 49. A board-certified veterinary ophthalmologist conducted the examinations. The animals were sedated with ketamine and tropicamide was instilled in each eye. Slit lamp biomicroscopy was used to examine the anterior segment, lens, and anterior vitreous. The anterior segment was scored using the modified Hackett McDonald scale.^30^ Indirect ophthalmoscopy was used to examine the posterior segment, including vitreous, fundus, and optic disc. Tonometry was performed following pharmacologic mydriasis using a TonoVet^®^ tonometer (Icare, Vantaa, Finland) on the default setting under laboratory light conditions. Ophthalmic examinations and tonometry were performed at approximately the same time of day as when the IVT injections were performed. Topical anesthetic [0.5% proparacaine (Bausch & Lomb)] was applied to the ocular surface before tonometry, and tobramycin [0.3% ophthalmic solution (Wintac, Ltd., Bangalore, India)] was applied to the eyes after tonometry.

### Electroretinograms

The ERG test consisted of a series of five light sequences to measure scotopic and photopic responses (rod response, maximal response, oscillatory potential, cone response, and flicker response) using parameters for standard full-field clinical ERG, as presented by the International Society for Clinical Electrophysiology of Vision.^31^ Animals were fasted ≥2 h before ERG and dark adapted for ≥30 min before the scotopic tests. Animals were sedated and the pupils were dilated with tropicamide.

### Serum TK and ADA assays

#### Determination of bevacizumab-bvzr concentrations in serum

Samples were analyzed using validated enzyme-linked immunoassay (ELISA) with bevacizumab-bvzr captured using recombinant VEGF A. SureBlue™ 3,3′, 5, 5′-tetramethylbenzidine was utilized as the peroxidase substrate for signal generation and colorimetric readout. The resulting signal was directly proportional to the concentration of bevacizumab-bvzr. The lower limit of quantitation (LLOQ) in serum was 100 ng/mL (0.1 μg/mL).

#### Determination of ADA in serum

Samples were analyzed using a Meso Scale Discovery platform (Meso Scale Discovery, Rockville, MD). Bevacizumab-bvzr was labeled with biotin and ruthenium, and acid dissociation was used to disassociate the drug–antibody complexes. An antibody-bridge results in the binding of the biotinylated and ruthenylated bevacizumab-bvzr to ADA. Samples with relative light unit (RLU) below the assay cut point were negative. Samples with RLU above the assay cut point were reanalyzed in a full dilution series to determine the antibody titer. The antibody titer was defined as the reciprocal dilution of the sample that would generate an RLU equal to the cut point RLU. The induction of ADA was based on the comparison of samples collected before dosing at Week −2 and postdose sample results.

### Tissue collection and preservation

#### Collection of vitreous humor and ocular tissues for bioanalysis

VH, C/RPE, and retina were collected at the time of necropsy from a subset of Group 1 control animals on Days 30 (dosing phase necropsy) and 57 (recovery phase necropsy) and of Group 3 animals each on Days 30, 39, and 57.

Each ocular component collected was weighed, placed in a separate tube, frozen immediately in liquid nitrogen, and transferred to a freezer (−80°C). VH for each eye was collected as a single aliquot per eye (i.e., VH between eyes were not pooled), frozen immediately in liquid nitrogen, and transferred to a freezer (−80°C). Concentrations of bevacizumab-bvzr in ocular samples were analyzed using the following validated methods.

#### Choroid/retinal pigment epithelium homogenate and retina tissue processing for bioanalysis

Bevacizumab-bvzr concentrations in C/RPE and retina homogenate were reported in ng per mL of tissue homogenate and in ng per mg of tissue. Before analysis, the tissue samples were processed using 1 × Halt Protease Inhibitor (ThermoFisher Scientific, Waltham, MA) in IP Lysis Buffer (Boster Bio, Pleasanton, CA) and homogenized using a QIAGEN TissueLyser II (QIAGEN GmbH, Hilden, Germany). The sample was centrifuged at 12,000 g for 10 min at 4°C, then chilled on ice for an additional 10 min. The supernatant was transferred into a new tube and additional homogenization buffer was added to bring the final amount buffer to 10.0 mg per 1 mL of tissue and then stored at −70°C.

### Determination of bevacizumab-bvzr tissue concentrations

VH, C/RPE homogenate, and retina homogenate were analyzed for bevacizumab-bvzr concentrations using validated ELISA methods (including precision/accuracy, dilution linearity, bench top stability, long-term stability, and freeze-thaw stability data). The LLOQ for VH, C/RPE homogenate, and retina homogenate were 100 ng/mL, 100 ng/mL (10 ng/mg), and 100 ng/mL (10 ng/mg), respectively. Tissue samples were not analyzed for ADA.

### TK calculations

All TK parameters were determined from individual animal data using noncompartmental analysis in Phoenix^®^ WinNonlin^®^ software (version 6.3; Certara, Princeton, NJ) using nominal times. Actual sampling times were used for Day 57 samples as there was no nominal time for the Day 57 sample. The area under the serum concentration-time curves AUC_24_, AUC_168_, AUC_336_, and AUC_tlast_ were estimated using the linear trapezoidal rule. For TK calculations, concentrations of 0 μg/mL were used for all results of below the limit of quantification (BLQ; <LLOQ of 100 ng/mL).

### Histology and microscopic evaluation

At necropsy, both eyes with optic nerves attached were collected from all animals and preserved in Davidson's fixative. The eyes collected for histology of Group 1 and Group 2 animals were routinely processed to paraffin and sectioned parallel to the ciliary artery to include optic nerve, macula, fovea, and optic disc. After the section was faced, five 5 μm sections (step-sectioned at ∼30-μm steps) were collected for microscopic evaluation (hematoxylin and eosin stained). Board-certified veterinary pathologists carried out the microscopic evaluation and peer review.

### Statistical analysis

Means and standard deviations were calculated for tonometry, ERG parameters, and body weights. Statistical analysis was performed on IOPs, body weights, and ERG data. Levene's test was used to assess the homogeneity of group variances parametric assumption at the 5% significance level. Datasets with at least 3 groups were compared using an overall one-way analysis of variance *F* test or Kruskal–Wallis test (if parametric assumptions were not met) at the 5% significance level. The above pairwise comparisons were conducted using a two-sided Dunnett's or Dunn's test, respectively, if the overall test was significant. All significant pairwise comparisons were reported at the 1 and 5% significance levels.

## Results

Repeat-dose IVT injection of bevacizumab-bvzr at 1.25 mg/eye/dose to cynomolgus monkeys was tolerated locally and systemically. No bevacizumab-bvzr-related mortality, effects on body weights, qualitative food consumption, IOP, or ERGs, or veterinary physical examination, macroscopic, or microscopic observations occurred in this study. IOP in all groups remained within normal ranges during the course of the study. Group 3 animals tended to have slightly lower pressures after dosing than Group 1 or Group 2. Group 3 prestudy pressure mean values were 18.0 mmHg right eye (OD) and 18.7 mmHg left eye (OS). Pressures beginning with Day 3 declined to mean values of 15.2 mmHg OD and 15.4 mmHg OS. Group 3 animal 3005 had the lowest individual pressure of 10 mmHg coinciding with the observed diffuse hyperemia of the conjunctivae, and this contributed to the lower mean IOP in the Group.

The general range of normal variability in ERG results in NHPs is ±25% of predose ERG. The Group mean ERG values were all within the normal range. Individual animal ERG values that were lower than the normal range included the following: Group 2 animal 2002 had oscillatory potential OD 73.5% and OS 109.2% of predose value; Group 3 animal 3005 had oscillatory potential OD 66.0% and OS 81.1% of predose value; Group 3 animal 3006 had cone response OD 116.4% and 73.4% OS. Group 1 animals received 50 mL volume of 0.9 saline solution via IVT injection in the left eye and 50 mL volume of the control article via IVT injection in the right eye, ERG and tonometry values were within normal ranges for both eyes throughout the study.

Minimal findings were present in the eyes of monkeys administered saline, vehicle, and bevacizumab-bvzr. Findings were composed of minimal focal breaks with fibrosis in the sclera and choroid, and disarray/vacuolation of the overlying retina. The incidence was as follows (Day 30 and 57): saline: 1/3 and 0/1 eyes, control article: 1/3 and 0/1 eyes, and bevacizumab-bvzr: 4/8 and 1/4 eyes. There were no optic nerve findings.

Trace pigment and cells were observed in the vitreous of 1 or both eyes during ophthalmic examinations in 4 of the 12 animals. These findings were first noted on Day 17 (2 days after the second dose) in some animals and only in bevacizumab-bvzr-dosed eyes. There were no microscopic correlates for these ophthalmic examination observations. The presence of pigment/cells in the vitreous is a common finding associated with IVT injection^32^; however, it was not noted in any concurrent control animals and was therefore considered bevacizumab-bvzr-related but not adverse.

On Day 3 of the dosing phase, there was a single clinical observation of eyeball redness at bevacizumab-bvzr 1.25 mg/eye/dose. The same animal had nonadverse, bevacizumab-bvzr-related conjunctival congestion and swelling bilaterally (2+ and 1+, respectively, as per Hackett and McDonald, 1996^30^), and diffuse bilateral conjunctival hyperemia on Day 3 following the first dose. These findings were again present at Days 17 and 31, with congestion showing signs of resolution (1+) and swelling remaining mild (1+); and conjunctival hyperemia was scored as very mild. The ocular inflammation was resolved by Day 39 (10 days after the last dose).

Slight, transient, decreased IOP (generally within the range of baseline values) in this animal was considered unrelated to bevacizumab-bvzr. Considering that these observations were transient, resolved with minimal treatment (tobramycin eyedrops up to twice daily on Days 4 through 8 in addition to the 2-day administration as part of the IVT injection dosing procedure), and there were no bevacizumab-bvzr-related microscopic findings at its scheduled necropsy on Day 57, these findings were considered nonadverse.

There were no bevacizumab-bvzr-related findings upon microscopic evaluation at the end of the dosing and recovery phases, resulting in a no observed adverse effect level (NOAEL) of 1.25 mg/eye/dose based on the totality of the results, mild severity of effects, lack of functional effects, and reversibility.

Administration of vehicle control or saline did not produce abnormalities during ophthalmic examinations. Transient fluctuations in IOP were considered unrelated to vehicle control or saline because they were generally within baseline fluctuations. At the end of the dosing and recovery phases, no microscopic findings of eyes or optic nerves or ERG readouts were associated with repeat-dose IVT injection of vehicle control or saline.

**FIG. 2.**
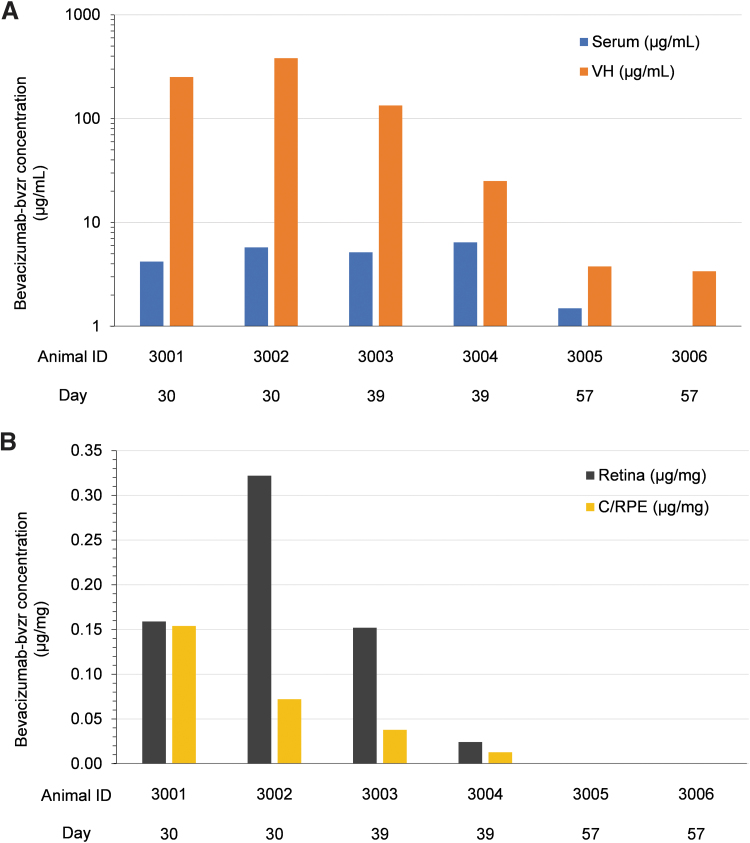
Bevacizumab-bvzr concentrations in **(A)** Serum and VH and **(B)** C/RPE and retina on Days 30, 39, and 57 in male cynomolgus monkeys administered bevacizumab-bvzr intravitreally at 1.25 mg/eye on Days 1, 15, and 29. On Day 57, serum bevacizumab-bvzr concentration was BLQ (< 0.1 μg/mL) for Animal 3006; retina and C/RPE bevacizumab-bvzr concentrations were BLQ (< 0.01 μg/mg) for Animals 3005 and 3006. BLQ, below the limit of quantification; C/RPE, choroid/retinal pigment epithelium; VH, vitreous humor.

Mean systemic C_max_ and AUC_336_ values were 5.1 μg/mL and 1490 μg·h/mL after 3 doses. Following repeated administration, there was evidence of accumulation of bevacizumab-bvzr in serum (based on mean AUC_336_: Day 15/Day 1 = 1.7; Day 29/Day 1 = 1.6) (Table 1). There were no quantifiable concentrations of bevacizumab-bvzr in samples collected and analyzed from the control group at any time point during the study or any bevacizumab-bvzr-dosed group before dosing on Day 1. The incidence of serum ADA induction to bevacizumab-bvzr was 100%, with ADA detected in all 12 bevacizumab-bvzr-dosed animals beginning by Day 8 following the first dose. Concentrations of bevacizumab-bvzr were measured in VH, retina, and C/RPE of bevacizumab-bvzr-treated animals on Days 30, 39, and 57. Following the Day 29 dose (third dose), bevacizumab-bvzr was quantifiable in the VH, retina, and C/RPE (Table 2 and Fig. 2a, b).

**Table 1.** Mean Toxicokinetic Parameters of Bevacizumab-bvzr in Serum of Male Cynomolgus Monkeys Following Repeat-Dose Intravitreal Injection of 1.25 mg/eye on Days 1, 15, and 29

Dose (mg/dose)	Day	C_max_ (μg/mL)			T_max_ (h)			AUC_24_ (μg·h/mL)			AUC_168_ (μg·h/mL)			AUC_336_ (μg·h/mL)			AUC_tlast_ (μg·h/mL)		
		Mean	SD	*n*	Mean	SD	*n*	Mean	SD	*n*	Mean	SD	*n*	Mean	SD	*n*	Mean	SD	*n*
2.5^a^ (Groups 2 and 3)	1	2.88	0.52	12	152	79	12	24	11	12	379	63	12	827	133	12	NC	NC	NC
	15	4.77	0.57	12	138	89	12	81	16	12	711	109	12	1420	197	12	NC	NC	NC
	29	5.10	1.60	4^b^	132	72	4^b^	107	19	12	831	232	6^c^	1490	631	4^d^	2390	1280	4^e^

^a^ 1.25 mg/OD (oculus dexter, or right eye) +1.25 mg/OS (oculus sinister, or left eye).

^b^ *N* = 4; C_max_ and T_max_ on Day 29 were calculated only for 4 recovery animals whose samples were collected up to Day 57.

^c^ *N* = 6; AUC_168_ was not calculated for 6 animals because those animals were euthanized on scheduled Day 30.

^d^ *N* = 4; AUC_336_ was not calculated for 8 animals because those animals were euthanized on scheduled Day 30 or 39.

^e^ AUC_tlast_ was calculated only on Day 29 for 4 recovery animals whose samples were collected up to Day 57 (∼668 h after dosing on Day 29).

AUC_24_, area under the concentration-time curve from time 0 to 24 h postdose; AUC_168_, area under the concentration-time curve from time 0 to 168 h postdose; AUC_336_, area under the concentration-time curve from time 0 to 336 h postdose; AUC_tlast_, area under the concentration-time curve from time 0 to the time of the last observed quantifiable concentration; C_max_, maximum observed concentration; n, sample size; NC, not calculated; SD, standard deviation; T_max_, time to first occurrence of C_max_.

**Table 2.** Concentrations of Bevacizumab-bvzr in Ocular Compartments of Male Cynomolgus Monkeys Following Repeat-Dose Intravitreal Injection of 1.25 mg/eye on Days 1, 15, and 29

Animal ID	Dose*^a^* (mg/eye)	Time point	Ocular compartment	Mean Conc. in compartment^b^	Mean Conc. in serum on day of eye collection (μg/mL)	Systemic exposures (serum) following the Day 29 dose			
						AUC_24_ (μg·h/mL)	AUC_168_ (μg·h/mL)	AUC_336_ (μg·h/mL)	AUC_tlast_ (μg·h/mL)
3001	1.25	Day 30	VH	252 μg/mL	4.20	98.7	NC^c^	NC^c^	NC
			Retina	0.159 μg/mg					
			C/RPE	0.154 μg/mg					
3002	1.25	Day 30	VH	383 μg/mL	5.75	134	NC^c^	NC^c^	NC
			Retina	0.322 μg/mg					
			C/RPE	0.072 μg/mg					
3003	1.25	Day 39	VH	134 μg/mL	5.16	102	811	NC^c^	NC
			Retina	0.152 μg/mg					
			C/RPE	0.038 μg/mg					
3004	1.25	Day 39	VH	25.1 μg/mL	6.43	126	1050	NC^c^	NC
			Retina	0.024 μg/mg					
			C/RPE	0.013 μg/mg					
3005	1.25	Day 57	VH	3.77 μg/mL	1.49	86.5	667	1340	2170
			Retina	BLQ					
			C/RPE	BLQ					
3006	1.25	Day 57	VH	3.39 μg/mL	BLQ	67.2	460	667	667
			Retina	BLQ					
			C/RPE	BLQ					

^a^ 50 μL of bevacizumab-bvzr dosing formulation was dosed by bilateral IVT injection (1.25 mg/eye) on Days 1, 15, and 29 to 6 animals (animal No: 3001, 3002, 3003, 3004, 3005, and 3006). Bevacizumab-bvzr was not detectable in serum or ocular compartments of Group 1 control animals during this study and therefore Group 1 data are not presented in this table.

^b^ Representative of the average concentration of bevacizumab-bvzr in each compartment of the left eye and right eye.

^c^ AUC_168_ and AUC_336_ were not calculated because animal was euthanized on scheduled Day 30 or 39.

AUC_24_, area under the concentration-time curve from time 0 to 24 h postdose, following the Day 29 dose; AUC_168_, area under the concentration-time curve from time 0 to 168 h postdose, following the Day 29 dose; AUC_336_, area under the concentration-time curve from time 0 to 336 h postdose, following the Day 29 dose; AUC_tlast_, area under the concentration-time curve from time 0 to the time of the last observed quantifiable concentration.

BLQ, below the limit of quantification (<100 ng/mL for serum; <100 ng/mL (<0.1 μg/mL) for VH; <10 ng/mg (<0.01 μg/mg) for C/RPE and retina); C/RPE, choroid/retinal pigment epithelium.

Conc., concentration; ID, identification number; IVT, intravitreal; NC, not calculated; VH, vitreous humor.

Mean concentrations of bevacizumab-bvzr were higher in VH relative to serum on Days 30, 39, and 57, and the VH:serum ratio of bevacizumab-bvzr gradually decreased between Days 30 and 57, with quantifiable bevacizumab-bvzr in serum of only 1 of the 2 Group 3 animals on Day 57. Mean concentrations of bevacizumab-bvzr in retina were higher than in C/RPE on Days 30 and 39, and BLQ (<10 ng/mg) in both tissues on Day 57. Under an assumption that the density of VH is similar to that of retina and C/RPE, the mean concentrations of bevacizumab-bvzr were in general higher in VH compared with retina and C/RPE at all 3 time points measured (Days 30, 39, and 57).

Systemic exposure (serum; Table 1 and Fig. 2a) to bevacizumab-bvzr increased with time among animals, which was consistent with the trend of decreased ocular concentrations of bevacizumab-bvzr through Day 57 and with bevacizumab-bvzr passing across the retinal-blood barrier into systemic circulation.

## Discussion

Repeat-dose IVT injection of bevacizumab-bvzr at 1.25 mg/eye/dose (once every 2 weeks, 3 doses total) was tolerated locally and systemically, and there were no bevacizumab-bvzr-related microscopic changes or effects on IOP or ERGs. Bevacizumab-bvzr-related trace pigment or cells in VH (commonly associated with IVT injection) in 4 of 12 animals and transient, nonadverse, and mild ocular inflammation in 1 of 12 animals were noted upon ophthalmic examination. There were no bevacizumab-bvzr-related clinical or ophthalmic observations during the 4-week recovery phase. The control article for bevacizumab-bvzr was also tolerated locally, and no changes to any ocular-related parameters were attributable to the control article. There were no control article-related microscopic changes. Based on these results, the NOAEL was 1.25 mg/eye/dose.

In repeat-dose ocular toxicity studies in cynomolgus monkeys of up to 6 months, bilateral IVT administration of Lucentis^21^ every 2 or 4 weeks at 0.25 to 2 mg/eye resulted in no systemic toxicity or effects in ERGs, but did result in dose-related inflammatory responses and appearance of cells and floaters in the vitreous at all doses in all studies. All inflammatory responses reversed or reduced following 4- to 8-week recovery periods. Immediate and marked increases (2–5 × ) in IOP were observed in control and Lucentis-dosed animals and returned to normal at the next measurement. Lower IOP was observed after Day 85 (7th dose) in a 6-month study at a dose of 2.0 mg/eye.^21^

For a closer comparison to this study with bevacizumab-bvzr, in a 4-week ocular toxicity study in monkeys with a 4-week recovery, bilateral IVT injection of Lucentis at 0.45 and 1.8 mg/eye (dose volume of 50 μL/eye) once every 2 weeks (total of 3 doses) resulted in dose-related, transient, and reversible inflammation of the eye characterized by the presence of cells, fibrin, and flare in the anterior chamber and accumulation of cells in the anterior vitreous.^21^ Transient retinal vasculitis, perivascular sheathing, vitreal opacity, and a tiny hemorrhage or microaneurysm were reported. Inflammatory cell infiltrates near the injection site were observed and considered related to IVT dosing procedure. At the recovery necropsy, the changes were minimal and of lesser severity, indicating that recovery was in process. No drug-related changes in IOP or ERGs were noted.^21^

The nonadverse, transient, and reversible mild ocular inflammation, characterized by eyeball redness, trace pigment and cells in the vitreous, conjunctival congestion, and swelling bilaterally with diffuse conjunctival hyperemia, observed following IVT injection of bevacizumab-bvzr in monkeys, is analogous and no greater in severity than that observed in the similarly designed study with Lucentis.^21^ Further, minimal focal breaks with fibrosis in the sclera and choroid, and disarray/vacuolation of the overlying retina, but not inflammatory cell infiltrates near the injection site, were occasionally noted on the Day 30 necropsy and less frequently on the Day 57 necropsy in eyes administered bevacizumab-bvzr, vehicle/control article, or 0.9% saline. Since the severity of these findings was the same and the incidence was similar across saline, vehicle, and bevacizumab-bvzr-dosed eyes, they were considered procedure-related and not bevacizumab-bvzr-related as in the Lucentis study.

Following IVT administration of bevacizumab (1.25 mg) to primates, signs of retinal damage, including induction of choriocapillaris abnormalities, were observed.^33^ In the present study with bevacizumab-bvzr, no microscopic effects were observed, including any effects on the retina, that were attributable to the administration of bevacizumab-bvzr.

Experiments in monkeys have confirmed that bevacizumab penetrates the retina to reach the site of action, with bevacizumab immunoreactivity in the choroid and the inner layers of the retina as early as 1 day after injection, thereafter spreading to the outer layers and the choroid within the following 7 days.^34^

In our current study, the mean systemic C_max_ and AUC_336_ values were 5.10 μg/mL and 1490 μg·h/mL after 3 doses, with evidence of accumulation. In addition, following the third dose, bevacizumab-bvzr was quantifiable in the VH, retina, and C/RPE. Under an assumption that the density of VH is similar to that of retina and C/RPE, the mean concentrations of bevacizumab-bvzr were highest in VH relative to retina and C/RPE, with mean concentrations of bevacizumab-bvzr among the 3 ocular compartments highest on Day 30, decreased by Day 39, and quantifiable only in VH on Day 57. Between Days 30 and 39, there was no clear trend between decreased bevacizumab-bvzr concentrations in ocular compartments and serum. However, systemic exposure (serum) to bevacizumab-bvzr increased with time across animals, which was consistent with the trend of decreased ocular concentrations of bevacizumab-bvzr through Day 57.

Serum ADA to bevacizumab-bvzr was detected in all 12 bevacizumab-bvzr-dosed animals beginning by Day 8 following the first dose. Quantifiable serum concentrations of bevacizumab-bvzr were observed until the last samples were collected for all dose groups except for 1 animal. In a previous study where monkeys were dosed bevacizumab-bvzr at 10 mg/kg/dose twice weekly intravenously for 1 month (total of 9 doses), no ADA induction was observed, although the high drug concentrations could have interfered with the detection of ADA as the drug levels at the time of ADA assessment were well above the drug tolerance limit for the ADA assay.^16^

Transient and mild IOP fluctuation was observed in certain animals after IVT administration, which was considered more physical rather than pharmacological effects. The cause of immediate IOP increase was likely due to sudden addition of test fluid to the enclosed vitreous cavity, and the pressure usually rebalanced and returned to normal shortly postinjection.

During the 4-week recovery phase, there were no biologically meaningful changes in IOP measurements. Therefore, this reversible IOP change appeared to be procedure-associated, and consistent with a well-known phenomenon that short-term IOP rise has been regarded as a fairly common and well-recognized effect after IVT administration in clinical setting with anti-VEGF treatment.^35^ Although the causes of long-term IOP increase were less clear, repeat intraocular injection and procedure-related inflammation could have contributed to IOP imbalance.^36^ In the current study, only short-term or transient IOP effects were observed; no indication of any persistent IOP rise was observed with the 1 month repeat-dose IVT administration of bevacizumab-bvzr.

## Conclusions

In summary, the mild ocular effects and safety of bevacizumab-bvzr, when administered intravitreally at a clinically relevant dose in monkeys and as assessed by clinical observations, ophthalmic examinations, IOP, ERGs, and histopathology, were consistent with that of its vehicle control and saline as well as with the results of studies conducted with other bevacizumab products and with Lucentis. Although several nonclinical studies have independently evaluated the pharmacokinetics, distribution, and/or safety of bevacizumab following IVT administration in monkeys,^33,34,37^ this study provides a comprehensive ocular safety and TK assessment for a bevacizumab product at a clinically relevant dose.

## Supplementary Material

Supplemental data

## Authors' Confirmation

All listed authors have met all authorship criteria defined by the International Committee of Medical Journal Editors.

## Editorial Support

Editorial support was provided by Shuang Li of Engage Scientific Solutions and was funded by Pfizer.
