# Supplementary material for: Ocular Safety and Toxicokinetics of Bevacizumab-bvzr (Zirabev), a Bevacizumab Biosimilar, Administered to Cynomolgus Monkeys by Intravitreal Injection
Source: J Ocul Pharmacol Ther. 2023 Apr 4;39(3):215–24. doi: 10.1089/jop.2022.0059 (PMC10079248; doi:10.1089/jop.2022.0059)
Supplement: Supplemental data [file Suppl_Data.pdf]

Ocular Safety and Toxicokinetics of Bevacizumab-bvzr (Zirabev®), a Bevacizumab Biosimilar, Administered to Cynomolgus Monkeys by Intravitreal Injection

Marjorie A. Peraza,[1] Susan Hurst,[2] Wenhu Huang[3], Bernard S. Buetow,[3] Andrew J. Lickteig[4], J. Dan Lavach,[4] Denzil F. Frost,[4] Margaret E. Collins,[4]* Rani S. Sellers,[5]* Diane Matsumoto Smith[3]

[1]Drug Safety Research and Development, Pfizer Inc, Cambridge, Massachusetts.
[2]Biomedicine Design, Pharmacokinetics, Dynamics, and Metabolism, Pfizer Inc, Groton, Connecticut.
[3]Drug Safety Research and Development, Pfizer Inc, San Diego, California.
[4]Charles River Laboratories, Inc, Reno, Nevada.
[5]Drug Safety Research and Development, Pfizer Inc, Pearl River, New York.
*Affiliation when the study was carried out.

Corresponding author: Marjorie A. Peraza, Drug Safety Research and Development, Pfizer Inc, 300 Technology Square, Cambridge, MA 02139, USA. Phone: 978-447-0317; E-mail: marjorie.peraza@pfizer.com

Supplementary Materials

Animals

The monkey was selected as the test species in this ocular toxicity study. Based on the precedented use of males in the development of bevacizumab-bvzr (Zirabev®), only male animals were used in the current study, as male or female monkeys were considered equally suitable/acceptable for the evaluation of ocular toxicity given the absence of sex differences in the nonclinical profile observed for Lucentis.

Male cynomolgus monkeys were group (up to 3) housed and maintained at 64–84º F (18–29º C) with a relative humidity of 30–70% on a 12-hour light/12-hour dark cycle and ≥10 air changes/hour. Monkeys were fed PMI Nutrition International Certified Primate Chow No. 5048 provided daily in size- and age-appropriate amounts; fruits and vegetables were provided as supplements at least 2–3 times per week. Reverse osmosis treated/ultraviolet irradiated municipal water was provided freely throughout the study.

Intravitreal (IVT) Injection

An eyelid speculum was used inserted to keep each eyelid open during the IVT injection dosing procedure and the globe was retracted. Using Betadine® (5% ophthalmic prep solution; Alcon, Geneva, Switzerland) and a cotton swab applicator, the injection site was swabbed and lightly blotted. The needle (27G) of the dose syringe was inserted through the sclera and pars plana approximately 4 mm posterior to the limbus. The needle was directed posterior to the lens and advanced into the mid-vitreous. Each eye received a single dose injection of 50 µL of saline, vehicle control, or bevacizumab-bvzr (1.25 mg). Injections were slowly administered into the mid-vitreous. Forceps were used to grasp the conjunctiva surrounding the syringe prior to needle withdrawal. The conjunctiva was held with the forceps during and briefly following needle withdrawal to prevent reflux. Following lid speculum removal, tobramycin (0.3% ophthalmic solution, 1–3 drops per eye, Wintac Ltd, Bangalore, India), a topical antibiotic, was dispensed onto each eye to reduce the potential for infection immediately following dosing and for 2 consecutive days after dosing. Sedation was reversed with

Antisedan® (atipamezole; 0.6 mg/kg, IM, Zoetis, Parsippany, NJ). The animals were returned to their home cages when sufficiently recovered from the sedation.

Additional Assay Information

Bioanalytical assays for serum and tissues

The GLP ELISA methods for quantitative determination of bevacizumab-bvzr in cynomolgus monkey serum, vitreous humor, retinal homogenate, and choroid/retinal pigment epithelium homogenate were validated in line with study-concurrent FDA and European Medicines Agency (EMA) bioanalytical methods guidance documents.[1,2] The method validations were evaluated by and met the criteria in regard to assay performance with respect to precision, accuracy, selectivity, dilution linearity, and stability. The overall method validation quality control (QC) performance (precision [P] and accuracy [A]) for the cynomolgus monkey bioanalytical assays were serum (P: ≤18.1%; A: −2.3% to 6.2%), vitreous humor (P: ≤6.9%; A: ≤−10.7% to −8.4%), retinal homogenate (P: ≤4.9%; A: −11.8% to −8.0%), and choroid/retinal pigment epithelium homogenate (P: ≤5.7; A: 1.6% to 8%).

Anti-drug antibody assay for serum

The GLP ECL method for the determination of anti–bevacizumab-bvzr antibodies (anti-drug antibodies [ADA]) in cynomolgus monkey serum was validated in line with FDA and EMA guidance documents available during the original assay development,[3-5] the update review,[6-8] and several literature publications.[9,10] The method validation was evaluated by and met the criteria in regard to assay performance with respect to precision, accuracy, selectivity, drug tolerance, pro-zone effect, and stability. The

precision for the low positive control (LPC: 50 ng/mL) and the high positive control (HPC: 6000 ng/mL) were 4.1% (LPC) and11.9% (HPC) for intra-assay precision, and 7.8% (LPC) and11.3% (HPC) for inter-assay precision.

Reference Materials and Critical Reagents

The control individual normal and pooled normal cynomolgus monkey serum as well as hemolyzed serum were sourced from BioIVT (formerly Bioreclaimation IVT). The cynomolgus monkey vitreous humor, retina, and choroid/retinal pigment epithelium were sourced from BioIVT and Pfizer, Inc. The capture/coat ELISA reagent (recombinant human $VEGF_{165}$: 293-VE-050) was sourced from R&D Systems. The detection antibody ELISA reagent (Human IgG-heavy and light chain monkey-adsorbed antibody-HRP conjugate: A80-319P) was sourced from Bethyl Laboratories Inc., Montgomery, TX.

For the cynomolgous monkey serum ADA assay, the positive control (Affinity Purified Rabbit Polyclonal antibody against bevacizumab) was produced by Pfizer, Inc. and the negative control (Normal Pooled Cynomolgus Monkey Serum – pooled from 10 individuals, 5 males/5 females) was sourced from BioIVT. Biotinylated and Ruthenylated bevacizumab-bvzr were prepared by QPS, LLC (Newark, DE).

REFERENCES

1. U.S. Food and Drug Administration. FDA Guidance for Industry: Bioanalytical Method Validation. May 2018. https://www.fda.gov/files/drugs/published/Bioanalytical-Method-Validation-Guidance-for-Industry.pdf (accessed September 20, 2022).

2. European Medicines Agency. EMA Guideline on Bioanalytical Method Validation. July 21, 2011. https://www.ema.europa.eu/en/documents/scientific-guideline/guideline-bioanalytical-method-validation_en.pdf (accessed September 20, 2022).

3. U.S. Food and Drug Administration. FDA Draft Guidance for Industry, Assay Development and Validation for Immunogenicity Testing of Therapeutic Protein Products. April 2016. https://www.fda.gov/files/drugs/published/Assay-Development-and-Validation-for-Immunogenicity-Testing-of-Therapeutic-Protein-Products.pdf (accessed September 20, 2022).

4. European Medicines Agency. EMA Guideline on Immunogenicity Assessment of Biotechnology-Derived Therapeutic Proteins. December 13, 2007. https://www.ema.europa.eu/en/documents/scientific-guideline/guideline-immunogenicity-assessment-biotechnology-derived-therapeutic-proteins-first-version_en.pdf (accessed September 20, 2022).

5. European Medicines Agency. EMA Guideline on Development, Production, Characterisation and Specifications for Monoclonal Antibodies and Related Products. December 18, 2008. https://www.ema.europa.eu/en/documents/scientific-guideline/guideline-development-production-characterisation-specifications-monoclonal-antibodies-related_en.pdf (accessed September 20, 2022).

6. U.S. Food and Drug Administration. Immunogenicity Testing of Therapeutic Protein Products — Developing and Validating Assays for Anti-Drug Antibody

Detection. January 2019. https://www.fda.gov/media/119788/download (accessed September 20, 2022).

7. European Medicines Agency. EMA Guideline on Immunogenicity assessment of therapeutic proteins. May 18, 2017. https://www.ema.europa.eu/en/documents/scientific-guideline/guideline-immunogenicity-assessment-therapeutic-proteins-revision-1_en.pdf (accessed September 20, 2022).

8. European Medicines Agency. EMA Guideline on Development, Production, Characterisation and Specifications for Monoclonal Antibodies and Related Products. July 21, 2016. https://www.ema.europa.eu/en/documents/scientific-guideline/guideline-development-production-characterisation-specification-monoclonal-antibodies-related_en.pdf (accessed September 20, 2022).

9. G. Shankar, V. Devanarayan, L. Amaravadi et al. Recommendations for the Validation of Immunoassays used for detection of host antibodies against biotechnology products. *J. Pharm. Biom. Analysis.* 2008; 48:1267-1281.

10. Pihl, S., Michaut, L, Hendricks, J., et al. EBF recommendation for stability testing of anti-drug antibodies; lessons learned from anti-vaccine antibody stabilities studies. *Bioanalysis.* 2014; 6:1409-1413.
